# Mapping key amino acid residues for the epimerase efficiency and stereospecificity of the sex pheromone biosynthetic short-chain dehydrogenases/reductases of *Nasonia*

**DOI:** 10.1038/s41598-018-37200-7

**Published:** 2019-01-23

**Authors:** Florian Semmelmann, John Hofferberth, Joachim Ruther, Reinhard Sterner

**Affiliations:** 1 Institute of Biophysics and Physical Biochemistry, University of Regensburg, D-93053 Regensburg, Germany; 2 Department of Chemistry, Kenyon College, Gambier, OH 43022 USA; 3 Institute of Zoology, University of Regensburg, 93053 Regensburg, Germany

## Abstract

Males of the parasitic wasp genus *Nasonia* use blends of chiral hydroxylactones as sex pheromones to attract conspecific females. Whereas all *Nasonia* species use a mixture of (4*R*,5*S*)-5-hydroxy-4-decanolide (RS) and 4-methylquinazoline (MQ) as sex pheromones, *Nasonia vitripennis* evolved (4*R*,5*R*)-5-hydroxy-4-decanolide (RR) as an extra sex pheromone component. We recently identified and functionally characterized three short-chain dehydrogenases/reductases (SDRs) NV10127, NV10128, and NV10129 that are capable of catalyzing the epimerization of RS to RR via (4*R*)-5-oxo-4-decanolide (ODL) as intermediate. Despite their very high sequence identities of 88–98%, these proteins differ drastically in their ability to epimerize RS to RR and in their stereoselectivity when reducing ODL to RR/RS. Here, in order to unravel the sequence differences underlying these varying functional properties of NV1027, NV10128 and NV10129, we created chimeras of the three enzymes and monitored their catalytic activities *in vitro*. The results show that a few amino acid changes at the C-termini and active sites of *Nasonia vitripennis* SDRs lead to substantially altered RS to RR epimerization and ODL-reduction activities. Thus, our study adds to the understanding of pheromone evolution by showing that subtle mutations in key biosynthetic enzymes can result in drastic effects on the composition of chemical signals.

## Introduction

Sex pheromones are pivotal for mate location, recognition, and acceptance in insects^1,2^. Evolutionary diversification of the underlying chemical signals can lead to the development of exclusive channels for sexual communication and thus minimize the likelihood of non-productive sexual interactions^3^. The genetic and biochemical mechanisms underlying sex pheromone diversification, however, are poorly understood. Therefore, further work in insect models such as moths^4^, fruit flies^5^ or parasitic wasps^6^ is required to disentangle the patterns of sex pheromone diversification that are associated with reproductive isolation^7^. Additionally, the in-depth study of insect pheromone biosynthesis may enable general conclusions about the sequence-based structure-activity relationships of enzymes catalyzing ubiquitous conversions of natural products^8^.

**Figure 1:**
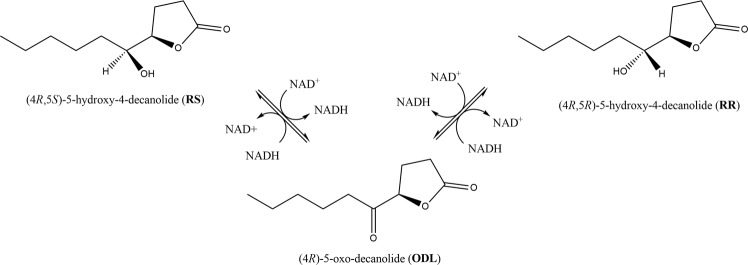
Putative epimerization mechanism of RS to RR via ODL by *Nasonia* SDRs. (4*R*,5*S*)-5-hydroxy-4-decanolide (RS) is epimerized to (4*R*,5*R*)-5-hydroxy-4-decanolide (RR) via (4*R*)-5-oxo-decanolide (ODL) as intermediate. NAD^+^/NADH act as cofactor.

The genus *Nasonia* consists of the four species *Nasonia giraulti* (*Ng*), *Nasonia longicornis* (*Nl*), *Nasonia oneida* (*No*), and *Nasonia vitripennis* (*Nv*)^9–11^. While males of all *Nasonia* species use blends of (4*R*,5*S*)-5-hydroxy-4-decanolide (RS) and 4-methylquinazoline (MQ) to attract conspecific females^12–14^, *Nv* evolved (4*R*,5*R*)-5-hydroxy-4-decanolide (RR) as an extra pheromone component^6,15^. The presence of RR allows virgin *Nv* females to discriminate against the less complex blend of other *Nasonia* species^6,16^. Genetic^6^ and biochemical^17^ analysis recently revealed a set of three highly similar short-chain dehydrogenases/reductases (SDRs) that catalyze the epimerization of RS to RR via (4*R*)-5-oxo-decanolide (ODL) as intermediate (Fig. 1). While all three SDRs, NV10127, NV10128, and NV10129 were capable of epimerizing RS to RR and reducing ODL to RR/RS, the enzymes varied drastically in their catalytic activities and stereospecificities *in vitro*. NV10128 was the most efficient at epimerizing RS to RR and stoichiometrically reduced ODL to RR and RS with a RR-biased product ratio^17^. NV10127, the most abundant enzyme in male *Nv* pheromone glands, was drastically less efficient at epimerizing RS to RR and was also capable of stoichiometrically reducing ODL in favor of the RR product. In contrast, NV10129 displayed RS to RR epimerization activity similar to that of NV10128, but the stereochemical outcome of ODL reduction was clearly RS-biased. The sequence and structural differences underlying the varying functional properties of NV1027, NV10128 and NV10129 remained unclear, though.

Here, as a first step to investigate the structure-activity relationship underlying *Nv* SDRs activity, we aimed to identify sequence motifs for the different functional properties of NV10127, NV10128, and NV10129. To this end, we used site-directed mutagenesis to generate chimeras of the three enzymes, heterologously expressed the corresponding genes in *Escherichia coli*, purified the proteins to homogeneity and monitored their catalytic activities *in vitro*. We identified C-terminal residues as key determinants of epimerization activities and amino acids in the active site that determine whether *Nv* SDRs reduce ODL to RR or RS. Our results thus show that subtle sequence variations of SDRs result in drastic functional consequences and provide novel insights in the sequence-related structure/activity relationships underlying the enzymatic epimerization of natural products.

## Results and Discussion

**Figure 2:**
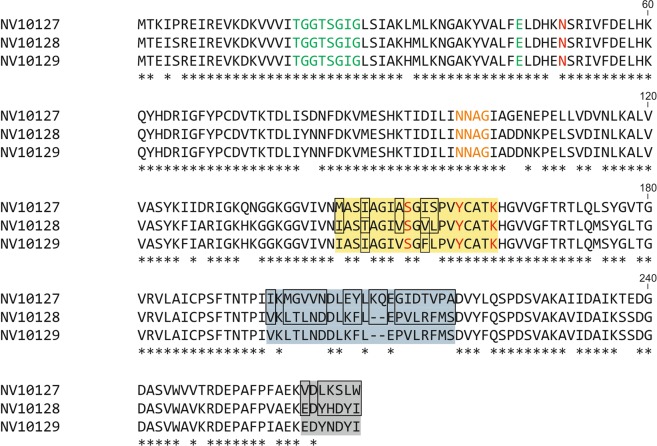
Multiple Sequence Alignment of *Nasonia vitripennis* SDRs investigated in this study. Sequence identities between NV10127 and NV10128/NV10129 are 214/266 (88.4%)/ 215/266 (88.8%) and between NV10128 and NV10129 are 262/266 (98.5%). Catalytically active amino acid residues are shown in red, amino acids involved in NAD^+^ binding are shown in green, and amino acids stabilizing the central β-sheet are shown in orange. Regions with high amino acid variation are highlighted in yellow (active site), light blue (sequence stretch downstream the active site), and light grey (C-terminal residues). The boxes in the alignments are the sites at which mutations were performed. Conserved amino acids are indicated by asterisks at the bottom of each column.

To unravel the sequence variations between NV10127, NV10128, and NV10129 that are responsible for the differences in RS to RR epimerization efficiency as well as the stereoselectivity of ODL-reduction, we generated a multiple sequence alignment (MSA) for the three enzymes (Fig. 2; sequences taken from^6^).

NV10127-29 are classical short-chain dehydrogenases (SDRs) *in sensu* Persson *et al*.^18^. They contain 266 (NV10127) and 264 (NV10128/29) amino acids and share a common Rossman fold topology with a conserved _19_TGGTSGIG_26_ motif for NAD^+^/NADH binding^19^ and a catalytic tetrad composed of N_51_-S_152_-Y_158_-K_162_^18^. The preference for NAD^+^ over NADP^+^ as coenzyme was predicted to be caused by E_45_^18^ and a conserved _98_NNAG_101_ motif is supposed to stabilize the central beta-sheet^20^. After heterologous expression in an *Escherichia coli* expression system and purification by affinity chromatography, the purified enzymes formed stable and homogeneous dimers in analytical gel filtration experiments (SI Fig. 1) and displayed melting temperatures between 45 and 55 °C in unfolding measurements monitored by nano differential scanning fluorimetry (SI Table 1).

**Figure 3:**
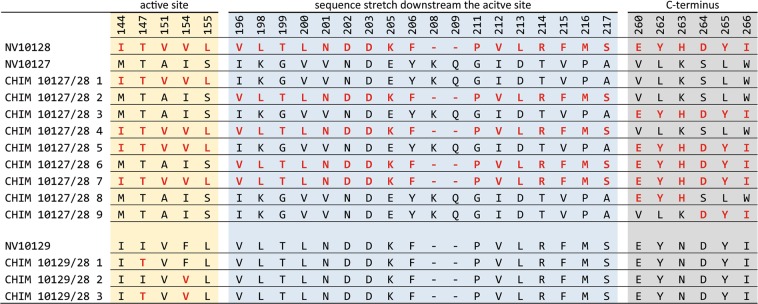
Overview of chimeras generated and characterized in this study. Amino acid sequence of regions with high variations of NV10127, NV10128, NV10129 and generated chimeras are shown in single letter code. The amino acids transferred from the reference sequence (NV10128, top row) into the scaffolds of NV10127 and NV10129 are shown in bold red. Wild-type amino acids of NV10127 and NV10129 are shown in standard black.

In order to explain the drastically lowered RS to RR epimerization activity of NV10127 compared to NV10128/29, we used NV10128, which was the most efficient enzyme in our *in vitro* epimerization assays^17^, as reference in a gain-of-function approach. By comparing the amino acid sequences of NV10127 and NV10128 we found a total of 50 amino acid differences (30 non-conservative and 20 conservative) along with a short gap in NV10128. Interestingly, most of these differences clustered: five were located in the active site, 17 in a sequence stretch beginning 33 amino acids downstream the active site and six at the C-terminal end. The remaining 22 differences were scattered across the whole sequence (Fig. 2). To keep the overall number of constructs as low as possible, we created chimeras by PCR-based site-directed mutagenesis thereby simultaneously replacing multiple cluster residues of NV10127 with corresponding residues of NV10128. Specifically, we generated chimeras by transferring amino acids from (i) the active site, (ii) the sequence stretch beginning 33 amino acids downstream the active site, (iii) the C-terminal end, as well as all possible combinations of (i)-(iii) (Fig. 3, CHIM NV10127/28 1–7).

In order to explain the clearly RS-biased ODL reduction of NV10129 compared to the clearly RR-biased ODL reduction of NV10127/28, we again used NV10128 as reference sequence in a gain-of-function approach. By comparing the amino acid sequence of NV10129 with the reference sequence NV10128 we found two non-conservative variations in the active site and two conservative variations at the C-terminal ends (Fig. 2). We created chimeras by transferring the two non-conservative mutations (I147T and F154V) into the active site from NV10128 into NV10129, both individually and combined, (Fig. 3, CHIM NV10129/28 1–3).

We expressed the genes of the mutated forms of NV10127 and NV10129 heterologously in *E. coli* and purified the enzymes to homogeneity. We then supplemented each of the mutants with saturating concentrations of NADH or NAD^+^ as well as with ODL or RS as substrates. After 1, 5, and 22 h we extracted subsamples with dichloromethane and followed ODL reduction or RS to RR epimerization activities of the enzymes by quantifying the amount of RR/RS/ODL in the extracts by GC/MS.

**Figure 4:**
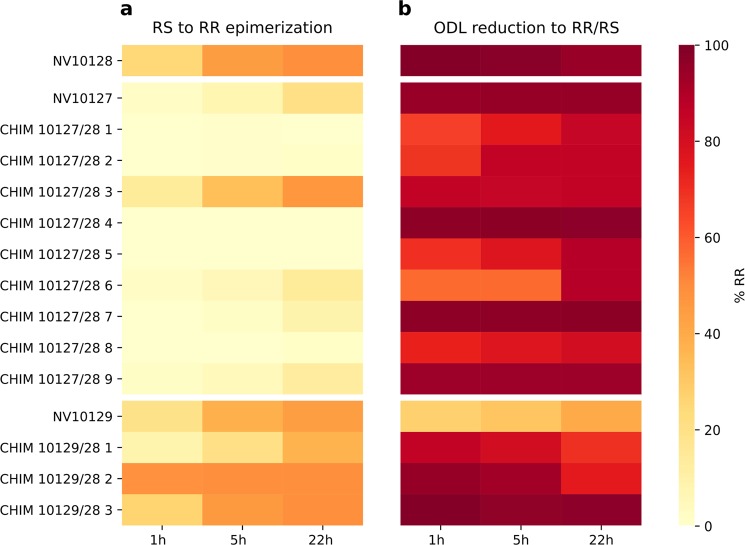
Heat maps indicating enzyme activities of NV10127-29 and chimeras. Percentage of RR formed by NV10127-29 and chimeras after addition of (**a**) 0.5 mM RS and (**b**) 0.5 mM ODL in the presence of saturating concentrations of NAD^+^ and NADH, respectively. In the reduction assays, ODL was almost stoichiometrically reduced to either RS or RR. Exact values with SDs are provided in SI Table S2.

The results of the functional assays showed that most chimeras with active site residues of NV10128 transferred into NV10127 were unable to epimerize RS to RR while the stereoselectivity of ODL-reduction was unchanged (Fig. 4). However, when only six amino acids of the C-terminal ends were swapped from NV10128 to NV10127 (CHIM NV10127/28 3), epimerization rates increased 7-fold, nearly reaching the rates observed for NV10128. Partial transfer of C-terminal residues (CHIM NV10127/28 8-9) diminished RS to RR epimerization rates, indicating that the entire C-terminal sequence is important for activity. Strikingly, neither the chimera containing the combination of mutations at the C-terminal end and the active site (CHIM NV10127/28 5) nor the chimera containing the combination of mutations at the C-terminal end, the active site, and the sequence stretch 33 amino acids downstream the active site (CHIM NV10127/28 7) was able to epimerize significant amounts of RS to RR. However, these chimeras readily reduced ODL to RR and there was no indication for misfolding or aggregation from analytical gel filtration and thermal denaturation experiments (SI Table 1). We conclude that the transferred active site residues of NV10128 generate a reaction center within the NV10127 scaffold that is non-productive for epimerization but still allows for a certain degree of ODL reduction activity. The latter is augmented by C-terminal residues of NV10128 that might either be directly involved in catalysis or contribute to a catalysis-competent active site configuration, for instance through proper substrate positioning. C-terminal ends might thereby act either within or between subunits (active site sharing) of homo-dimeric SDRs.

For the differences between NV10129 and NV1028 in the stereoselectivity of ODL-reduction, we found that both mutations in the active site of NV10129 individually (CHIM NV10129/28 1 and CHIM NV10129/28 2) as well as combined (CHIM NV10129/28 3) shifted the RS-biased ODL reduction to clearly RR-biased ODL reduction. As the I147T mutation in NV10129 (CHIM NV10129/28 1) only affected stereoselectivity of ODL reduction but not RS to RR epimerization activity, it is plausible to assume that the newly introduced side chain stabilizes the transition state leading from ODL to RR through polar interactions with its hydroxyl moiety. Interestingly, the F154V mutation in NV10129 (CHIM NV10129/2 2) not only altered the stereo-selectivity of ODL reduction almost stoichiometrically to RR but also accelerated the RS to RR epimerization rate, as indicated by an abundance of 48.4% RR (compared to 25.6% RR for the reference enzyme NV10128) after a reaction time of only 1 h (SI Table S2). We therefore suggest that steric constraints are released by the introduction of the smaller side chain (valine compared to phenylalanine), accelerating both oxidation of RS and reduction of ODL activities. By allowing greater substrate mobility, this mutation might facilitate the proposed rotation of substrates within the active site environment leading to the presentation of the opposite side of the substrate to the NAD^+^/NADH cofactor (Fig. 1)^17^. Combination of mutations I147T and F154V in NV10129 (CHIM NV10129/28 3) led to epimerization rates and stereo-selectivity of ODL reduction as observed for the reference enzyme NV10128.

## Conclusion

The NV10127-29 enzymes investigated in this study have sequence identities of 88–98% and the data obtained with the chimeras confirm that the evolution of new functional properties can be elicited by very small changes in the coding sequence of *N. vitripennis* SDRs. Together with the fact that the three SDRs are located physically close to each other on scaffold 1 of chromosome 1^6^ this suggests that NV10127-29 share a common SDR ancestor from which they have diverged by gene duplication and diversification. This ancestor might have been a 15-hydroxyprostaglandin dehydrogenase, a class of SDRs which deactivate prostaglandins and show significant sequence similarity of more than 60% to the *Nasonia* SDRs (SI Fig. 2). This hypothesis is furthermore supported by the fact that 15-hydroxyprostaglandin shares some striking structural similarities with RR/RS^6^. In insects as well as in other invertebrates and mammals, prostaglandins and other eicosanoids are involved in numerous crucial processes such as immune responses or the induction of egg-laying behavior^21^ (and references therein). Thus, it would be interesting to investigate if *Nasonia* SDRs ‘still’ accept 15-hydroxyprostaglandin as substrate or, if no promiscuous activities can be detected, if prostaglandin deactivation activity can be established through random mutagenesis at the key sites reported here.

Our observation that new functional properties can be elicited by very small changes within the coding sequences of *Nasonia* SDRs is in accordance with recent studies on moths suggesting that enzymes involved in sex pheromone diversification can evolve by duplication and neo-functionalization from already existing genes by as little as a single amino acid exchange^8^. Work on the sex pheromone composition of *Manducta sexta* has revealed that gene duplication of a fatty-acyl desaturase gene followed by small changes in its coding sequence are sufficient to result in the acquisition of novel sex pheromone components^8^. Also from the receivers’ perspective, pheromone communication systems can evolve rapidly. Work on *Ostrinia* moths has demonstrated that a single mutation in a duplicated sex pheromone receptor can change an entire pheromone recognition pattern^22^.

The presented data also demonstrate that residues classically assigned to the active site in canonical SDRs are not sufficient to explain the observed functional differences of *Nv* SDRs. In fact, we found that in the enzymes studied here six amino acid residues at the C-terminal ends are crucial for catalysis.

Interestingly, studies on UDP-4-sugar epimerases, the best studied SDRs with epimerase activity to date^23–26^, revealed striking similarities to the SDRs investigated here. While active site residues and cofactor binding sites are highly conserved in UDP-4-sugar-epimerases and *Nv* SDRs, C-terminal domains (UDP-4-sugar-epimerases) and C-terminal ends (*Nv* SDRs) vary drastically in both systems and were found to account for differing substrate specificities. Two models were subsequently developed for UDP-4-sugar-epimerases to explain the observed substrate specificities: the *hexagonal box* model^27^ and the *belt* model^23^. The hexagonal box model describes the active site of UDP-4-sugar-epimerases as a limited number of residues predominantly contributing to substrate specificity, much like a hexagonal box, where each wall (residue) defines the accessibility of the active site by the simple chemical nature of its side chain. The belt model, in contrast, describes a sequence stretch of residues as determinant of substrate specificity, much like a belt closing the active site environment. With the presented data, however, it is difficult to assign a model to the SDRs investigated in this study. Instead, a delicate balance between classical active site residues and complementary sequence stretches located at the C-terminus seem to determine substrate specificities and stereoselectivities of NV10127-29. In order to disentangle this sophisticated network of essential residues, a crystal structure of the enzymes studied in an abortive complex with coenzyme and substrate will ultimately be needed.

## Methods

### Cloning and mutagenesis

The genes of NV10127-29 were optimized for expression in *E. coli*, synthesized (Life Technologies) and cloned into pET28a_*BsaI* as described elsewhere^28^. Chimeras of NV10127/28 1–9 were generated according to the New England Biolabs Q5 Site-Directed Mutagenesis Kit using the oligonucleotides listed in SI Table 3. Chimeras NV10129/28 1–3 were generated using a modified QuickChange mutagenesis protocol^29^ with the primers listed in SI Table 3.

### Expression and purification of proteins

Proteins were produced by gene expression in *E. coli* BL21-Gold (DE3)-RIPL cells. Overnight cultures of individual clones were used to inoculate 1 L of Luria broth medium supplemented with 150 µg/mL chloramphenicol and 150 µg/ml kanamycin. Cells were grown at 37 °C to an OD_600_ of 0.6 and then cooled to 25 °C. Expression was induced by adding 0.5 mM isopropyl β-D-1-thiogalactopyranoside and growth was continued overnight at 20 °C. Cells were harvested by centrifugation (2700 g, 4 °C), suspended in 50 mM Tris-HCl, pH 7.5, 300 mM potassium chloride, 10 mM imidazole, and lysed by sonification. The insoluble fraction was removed by centrifugation (23000 g, 4 °C) and the soluble extracts were filtered through a 0.8 µm membrane.

Supernatants containing the *N*-terminal hexahistidine-tagged proteins were loaded onto a HisTrapFF crude column (5 mL, GE Healthcare), which had been equilibrated with suspension buffer, and eluted from the column by applying a linear gradient of 10–750 mM imidazole. Enzyme-containing fractions, as judged by SDS-PAGE, were pooled and further purified by preparative gel filtration (Superdex 75 HiLoad 26/60, 320 mL, GE Healthcare, 100 mM Tris-HCl, pH 7.5, 50 mM KCl, 4 °C). Elution fractions were analyzed by SDS-PAGE and the fractions containing pure protein were pooled. The enzymes were finally concentrated to 75–150 µM and flash frozen in liquid nitrogen. Protein concentrations were determined by measuring the absorbance at 280 nm, using the molar extinction (SI Table 4) coefficient calculated via ExPASy ProtParam (http://web.expasy.org/protparam/).

### *In vitro* epimerization and reduction assay with purified SDRs

A standard assay contained 5 µM of each protein, 1.5 mM NAD^+^ or NADH and 500 µM of RS or ODL in 100 mM TRIS HCl pH 7.5 and 50 mM KCl. When ODL was used as a precursor (reduction assay) NADH was employed alone as coenzyme. When RS was used as precursor (epimerization assay) NAD^+^ was employed as coenzyme.

The reaction mixtures were kept at 30 °C, shaken at a rate of 300 rpm, and samples of 250 µl each were taken after 1 h, 5 h and 22 h. All samples were extracted twice in 250 µl dichloromethane and analyzed by GC/MS. Each combination of protein/precursor/coenzyme/time was tested 3 times. Control assays were conducted for each treatment as described above without adding the proteins.

### Differential Scanning Fluorimetry (nanoDSF)

Purified proteins (100 µM concentration in 50 mM TRIS 7.5, 50 mM KCl) were heated in a Prometheus NT.48 (NanoTemper, Munich) from 20–95 °C at a ramp rate of 1 °C min^−1^ at the facilities of 2bind GmbH (Regensburg). The excitation power at 280 nm was 15% and emission spectra were recorded at 330 nm and 350 nm. The change in the ratio of the fluorescence signal from 350 to 330 nm with increasing temperature was fitted with PR.ThermControl (v2.0.3). The apparent midpoint temperatures (T_M_^app^) of the irreversible unfolding transition, defined by the inflection points of the curves, were determined as an operational measure of protein stability. Proteins were assayed in triplicates.

### GC/MS analysis

Chemical analyses were performed using a Shimadzu QP2010 Plus GC/MS system equipped with a 30 m × 0.25 mm inner diameter BPX5 capillary column (film thickness 0.25 µm, SGE Analytical Science Europe, Milton Keynes, UK). Samples (1 µl) were injected splitless at 300 °C using an AOC 20i auto sampler. Helium was used as carrier gas at a constant flow rate of 2 ml/min. The GC temperature program started at 80 °C and was programmed to increase at 5 °C/min to 280 °C and hold at this temperature for 15 min. Identification of compounds was accomplished by comparison of retention times and full scan mass spectra (mass range 35–500) with those of the synthetic reference compounds.

For analysis of the epimerization rates, we related the peak area of the epimerization product RR to the summarized peak areas of the unreacted RS and the product (RR + RS = 100%). For analysis of the reduction assay with ODL as precursor, we related the peak area of *RR* to the total peak area of both products (*ODL* + *RR* + *RS* = 100%).

### Analytical gel filtration

The oligomerization state of NV10127-NV10129 was analyzed by analytical size exclusion chromatography (SEC). Purified proteins (100 µM concentration in 50 mM TRIS 7.5, 50 mM KCl) were loaded on a Superdex 75 10/300 GL column (GE Healthcare) operated on an ÄKTAmicro system (GE Healthcare) connected to an ALIAS autosampler (Spark Holland). The system was operated at 25 °C with a flow rate of 0.5 mL/min of degassed buffer (50 mM Tris-HCl, pH 7.5, 50 mM KCl) and was calibrated with Conalbumin, Ovalbumin, Carbonic Anhydrase, Ribonuclease A and Aprotein from the GE Healthcare SEC Low-Molecular-Weight and High-Molecular-Weight calibration kits.

## Supplementary information

Supplementary Figures and Tables

## Data Availability

All data generated or analyzed during this study are included in this published article (and its Supplementary Information file), any further information is available from the corresponding author on reasonable request.
