# Supplementary material for: Mapping key amino acid residues for the epimerase efficiency and stereospecificity of the sex pheromone biosynthetic short-chain dehydrogenases/reductases of Nasonia
Source: Sci Rep. 2019 Jan 23;9:330. doi: 10.1038/s41598-018-37200-7 (PMC6344473; doi:10.1038/s41598-018-37200-7)
Supplement: Supplementary file 1 — Supplementary Figures and Tables [file 41598_2018_37200_MOESM1_ESM.pdf]

Supplementary Information

Mapping key amino acid residues for the epimerase efficiency and stereospecificity of the sex pheromone biosynthetic short-chain dehydrogenases/reductases of *Nasonia*

Florian Semmelmann[1], John Hofferberth[2], Joachim Ruther[3*], and Reinhard Sterner[1]

[1] Institute of Biophysics and Physical Biochemistry
University of Regensburg, D-93053 Regensburg, Germany

[2] Department of Chemistry
Kenyon College, Gambier, OH 43022, USA.

[3] Institute of Zoology
University of Regensburg, 93053 Regensburg, Germany.

[*]Corresponding author:
Joachim Ruther: +49-941 943 2151; Joachim.Ruther@ur.de

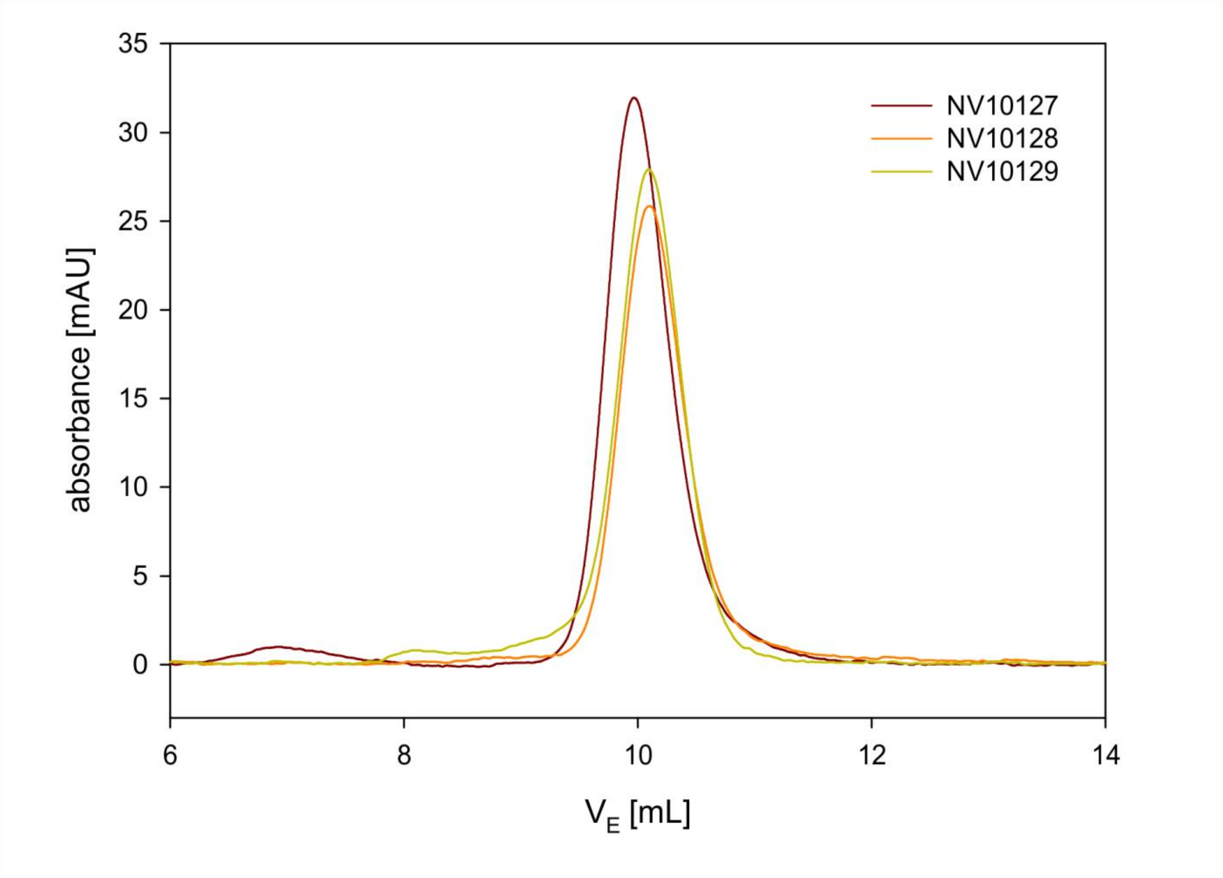

SI Figure 1. Elution profiles of analytical gel filtration runs of NV10127 (dark red), NV10128 (orange), and NV10129 (yellow).

```
                                                                            60
                                                                             |
MM_15-HPGD      M--------HVNGKVALVTGAAQGIGKAFAEALLLHGAK-VALVDWNLEAGVKCKAALDE
EC_15-HPGD      M--------HVNGKVALVTGAAQGIGRAFAEELLHKGAK-VALVDCNLEAGVKCKAALDE
CL_15-HPGD      M--------HVNGKVALVTGAAQGIGRASAEALLHKGAK-VALVDWNLEAGVKCKAALDE
NV10127         MTKIPREIREVKDKVVVITGGTSGIGLSIAKLMLKNGAKYVALFELDHKNSRIVFDELHK
NV10128         MTEISREIREVKDKVVVITGGTSGIGLSIAKHMLKNGAKYVALFELDHENSRIVFDELHK
NV10129         MTEISREIREVKDKVVVITGGTSGIGLSIAKHMLKNGAKYVALFELDHENSRIVFDELHK
                *        .*:.**.::**.:.*** : *: :* :*** ***.: : : .      *.:
                                                                           120
                                                                             |
MM_15-HPGD      QFEPQKTLFVQCDVADQKQLRDTFRKVVDHFGRLDILVNNAGVNNEKNWEQTLQINLVSV
EC_15-HPGD      QFEPRKTLFIQCDVANQEQLRDTFRKVVDHFGRLDILVNNAGVNNEKNWEKTLQINLVSV
CL_15-HPGD      QFEPQKTLFIQCDVADQGQLRDTFRKVVDHFGRLDILVNNAGVNNEKNWEKTVQINLVSV
NV10127         QYH-DRIGFYPCDVTKTDLISDNFDKVMESHKTIDILINNAGIAGENEPELLVDVNLKAL
NV10128         QYH-DRIGFYPCDVTKTDLIYNNFDKVMESHKTIDILINNAGIADDNKPELSVDINLKAL
NV10129         QYH-DRIGFYPCDVTKTDLIYNNFDKVMESHKTIDILINNAGIADDNKPELSVDINLKAL
                *:.  :  *  ***:.   : :.* **:: .  :***:****: .::: *  :::** ::
                                                                           180
                                                                             |
MM_15-HPGD      ISGTYLGLDYMSKQNGGEGGIIINMSSLAGLMPVAQQPVYCASKHGIIGFTRSAAMAANL
EC_15-HPGD      ISGTYLGLDYMSKQNGGEGGIIINMSSLAGLIPVAQQPVYCASKHGIIGFTRSAAMAANL
CL_15-HPGD      ISGTYLGLDYMSKQNGGEGGIIINMSSLAGLMPVAQQPVYCASKHGIIGFTRSAAMAANL
NV10127         VVASYKIIDRIGKQNGGKGGVIVNMASIAGIAS-GISPVYCATKHGVVGFTRTLQLSYGV
NV10128         VVASYKFIARIGKHKGGKGGVIVNIASTAGIVS-GVLPVYCATKHGVVGFTRTLQMSYGL
NV10129         VVASYKFIARIGKHKGGKGGVIVNIASIAGIVS-GFLPVYCATKHGVVGFTRTLQMSYGL
                : .:*  :  :.*::**:**:*:*::* **: . .  *****:***::****:  :: .:
                                                                           240
                                                                             |
MM_15-HPGD      MKSGVRLNVICPGFVDTPILESIEKEENMGQYIEYK--DQIKAMMKFYGVLHPSTIANGL
EC_15-HPGD      MNSGVRLNAICPGFVNTPILESIEKEENMGQYIEYK--DHIKDMMKFYGILDPSMIANGL
CL_15-HPGD      MNSGVRLNAICPGFVNTPILESIEKEENMGQYIEYK--DHIKDMMKFYGILDPSMIASGL
NV10127         --TGVRVLAICPSFTNTPIIKMGVVND-----LEYLKQEGIDTVPADVYLQSPDSVAKAI
NV10128         --TGVRVLAICPSFTNTPIVKLTLNDD-----LKFL--EPVLRFMSDVYFQSPDSVAKAV
NV10129         --TGVRVLAICPSFTNTPIVKLTLNDD-----LKFL--EPVLRFMSDVYFQSPDSVAKAV
                  :***: .***.*.:***::    ::     :::   : :  .     .  *. :*..:

MM_15-HPGD      INLIEDDALNGAIMKITASKGIHFQDYDISPLLVKAPLTS--
EC_15-HPGD      ITLIEDDALNGAIMKITTSKGIHFQNYDTIPFHTKTQ-----
CL_15-HPGD      ITLIEDDALNGAIMKITTSKGIHFQDYETTPFHAKTQ-----
NV10127         IDAIKTEDGDASVWVVT-------RDEPAFPFAEKVDLKSLW
NV10128         IDAIKSSDGDASVWAVK-------RDEPAFPVAEKEDYHDYI
NV10129         IDAIKSSDGDASVWAVK-------RDEPAFPIAEKEDYNDYI
                *  *: .  :.::  :.       ::    *.  *
```

SI Figure 2. Multiple Sequence Alignment of *Nasonia vitripennis* SDRs and functionally characterized 15-hydroxyprostaglandin dehydrogenases (15-HPGDs). 15-HPGD from *Mus musculus* (MM_15-HPGD, Accession Number: NP_032304.2), *Equus caballus* (EC_15-HPGD, Accession Number: ABC96794.1), *Canis lupus familiaris* (CL_15-HPGD, Accession Number: AFF60303.1) and the *Nasonia* SDRs studied here were aligned with MAFFT. Conserved amino acids are indicated by asterisks at the bottom of each column. Conservative differences are indicated by colons and dots at the bottom of each column.

SI Table 1. Melting temperatures of enzymes investigated in this study.

Protein	T_M^{app} [°C]
**NV10127**	45.0 ± 0.5
**NV10128**	47.4 ± 0,0
**NV10129**	54.8 ± 0.0
**CHIM NV10127/28 1**	53.2 ± 0.1
**CHIM NV10127/28 2**	42.1 ± 0.1
**CHIM NV10127/28 3**	49.8 ± 0.2
**CHIM NV10127/28 4**	42.8 ± 0.0
**CHIM NV10127/28 5**	47.0 ± 0.2
**CHIM NV10127/28 6**	38.6 ± 0.1
**CHIM NV10127/28 7**	48.9 ± 0.2
**CHIM NV10127/28 8**	46.4 ± 0.0
**CHIM NV10127/28 9**	44.7 ± 0.0
**CHIM NV10129/28 1**	48.8 ± 0.1
**CHIM NV10129/28 2**	47.2 ± 0.0
**CHIM NV10129/28 3**	42.2 ± 0.1

SI Table 2. Enzyme activities of NV10127-29 and chimeras. Percentage of RR formed by NV10127-29 and chimeras after addition of (**A**) 0.5 mM RS and (**B**) 0.5 mM ODL in the presence of saturating concentrations of NAD^+ and NADH, respectively. For the differences in RS to RR epimerization activity (NV10127, CHIM 10127/28 1-9, NV10128) each point in time was assayed 5 times and control assays of ODL reduction to RR/RS were assayed in duplicate. Analogously, for the differences in ODL to RR/RS reduction (NV10129, CHIM 10129/28 1-3, NV10128) each point in time was measured 5 times and control assays of RS to RR epimerization were assayed in duplicate.

**A**	**RS to RR epimerization**		
	1h	5h	22h
**NV10128**	25.3 ± 0.3	44.9 ± 0.1	48.9 ± 0.1
**NV10127**	2.2 ± 0.3	7.7 ± 0.4	20.2 ± 0.5
**CHIM 10127/28 1**	0.1 ± 0.0	0.4 ± 0.1	0.2 ± 0.0
**CHIM 10127/28 2**	0.1 ± 0.0	0.4 ± 0.0	1.4 ± 0.1
**CHIM 10127/28 3**	14.8 ± 0.7	33.3 ± 0.5	46.5 ± 0.4
**CHIM 10127/28 4**	0.0 ± 0.0	0.1 ± 0.0	0.2 ± 0.0
**CHIM 10127/28 5**	0. 1 ± 0.1	0.0 ±0.0	0.1 ± 0.0
**CHIM 10127/28 6**	2.0 ± 0.1	6.1 ± 0.3	14.8 ± 0.4
**CHIM 10127/28 7**	0.1 ± 0.0	1.6 ± 0.2	9.1 ± 0.8
**CHIM 10127/28 8**	0.0 ± 0.0	0.2 ± 0.1	0.9 ± 0.2
**CHIM 10127/28 9**	1.6 ± 0.1	4.9 ± 0.3	13.5 ±1.0
**NV10129**	19.5 ± 0.5	38.2 ± 0.1	44.2 ± 0.2
**CHIM 10129/28 1**	8.7 ± 0.9	21.0 ± 0.9	37.5 ± 1.5
**CHIM 10129/28 2**	48.5 ± 0.2	49.2 ± 0.2	49.1 ± 0.1
**CHIM 10129/28 3**	26.7 ± 0.3	46.0 ± 0.2	49.1 ± 0.1

**B**	**ODL reduction to RR [%]**					
	1h		5h		22h	
	ODL [%]	RR[%]	ODL [%]	RR[%]	ODL [%]	RR[%]
**NV10128**	0.2 ± 0.0	99.0 ± 0.1	0.2 ± 0.0	98.1 ± 0.2	0.2 ± 0.0	94.7 ± 0.5
**NV10127**	0.2 ± 0.1	94.7 ± 7.8	0.2 ± 0.0	95.6 ± 0.1	0.2 ± 0.0	95.3 ± 0.2
**CHIM 10127/28 1**	33.4 ± 0.2	66.4 ± 0.5	24.7 ± 1.5	75.3 ± 1.5	15.2 ± 0.5	84.8 ± 0.5
**CHIM 10127/28 2**	20.5 ± 1.9	68.8 ± 2.1	0.2 ± 0.0	86.1 ± 0.7	0.1 ± 0.0	85.9 ± 0.2
**CHIM 10127/28 3**	0.2 ± 0.0	86.1 ± 1.8	0.2 ± 0.0	85.1 ± 1.3	0.2 ± 0.1	85.9 ± 1.2
**CHIM 10127/28 4**	2.1 ± 1.1	96.7 ± 1.1	1.6 ± 0.8	97.0 ± 0.7	0.9 ± 1.0	97.5 ± 0.8
**CHIM 10127/28 5**	27.5 ± 12.0	70.0 ± 12.0	19.0 ± 9.0	77.6 ± 8.9	6.1 ± 3.4	89.0 ± 3.8
**CHIM 10127/28 6**	0.5 ± 0.5	56.8 ± 0.8	0.3 ± 0.0	56.7 ± 1.6	0.3 ± 55.6	89.0 ± 3.8
**CHIM 10127/28 7**	1.6 ± 2.0	96.7 ± 1.3	1.2 ± 1.6	96.5 ± 0.3	1.5 ± 0.2	96.9 ± 0.1
**CHIM 10127/28 8**	23.3 ± 13.9	73.7 ± 13.8	20.0 ± 12.8	76.9 ± 12.8	16.2 ± 10.8	80.6 ± 11.0
**CHIM 10127/28 9**	0.2 ± 0.1	94.0 ± 1.4	0.2 ± 0.1	94.1 ± 1.4	0.1 ± 0.1	94.1 ± 1.2
**NV10129**	0.2 ± 0.0	27.5 ± 0.4	0.2 ± 0.0	31.3 ± 0.2	0.2 ± 0.0	40.5 ± 0.2
**CHIM 10129/28 1**	0.2 ± 0.0	85.9 ± 0.2	0.2 ± 0.0	80.5 ± 0.6	0.2 ± 0.0	69.9 ± 5.0
**CHIM 10129/28 2**	0.2 ± 0.1	95.0 ± 0.1	0.2 ± 0.0	92.8 ± 3.0	0.2 ± 0.0	74.7 ± 5.5
**CHIM 10129/28 3**	0.2 ± 0.0	98.5 ± 0.0	0.3 ± 0.0	96.2 ± 2.9	0.2 ±0.0	97.6 ± 0.2

SI Table 3. Template DNA and oligonucleotides used for site-directed mutagenesis.

5’-phosphorylated oligonucleotides are marked with Ⓟ.

Gene	Template DNA	Primer (5′-3′)
**CHIM 10127/28 1**	pET28a_*Bsa*I_NV10127	Fo ⓅAGCCAGCGGAATCTCACCCGTTTACTGCGC<br>Re ⓅATGCCCGCGATTGATGCCATATTTACGATTACACC
**CHIM 10127/28 2**	pET28a_ *Bsa*I_NV10127	Fo ⓅTTGGAACCAGTTCTCAGATTCATGTCAGATGTTTACTTGCAGTCACCGGACAGTGTAGC<br>Re ⓅTTGGAACCAGTTCTCAGATTCATGTCAGATGTTTACTTGCAGTCACCGGACAGTGTAGC
**CHIM 10127/28 3**	pET28a_ *Bsa*I_NV10127	Fo ⓅGAAGATTATCATGATTATATTTGACTCGAGCACCACCACCACC<br>Re ⓅTTTTTCTGCAACTGGAAAAGCTGGCTCGTCTC
**CHIM 10127/28 4**	pET28a_ *Bsa*I_CHIM 10127/28 1	Fo ⓅTTGGAACCAGTTCTCAGATTCATGTCAGATGTTTACTTGCAGTCACCGGACAGTGTAGC<br>Re ⓅTTGGAACCAGTTCTCAGATTCATGTCAGATGTTTACTTGCAGTCACCGGACAGTGTAGC
**CHIM 10127/28 5**	pET28a_ *Bsa*I_CHIM 10127/28 1	Fo ⓅGAAGATTATCATGATTATATTTGACTCGAGCACCACCACCACC<br>Re ⓅTTTTTCTGCAACTGGAAAAGCTGGCTCGTCTC
**CHIM 10127/28 6**	pET28a_ *Bsa*I_ CHIM 10127/28 2	Fo ⓅGAAGATTATCATGATTATATTTGACTCGAGCACCACCACCACC<br>Re ⓅTTTTTCTGCAACTGGAAAAGCTGGCTCGTCTC
**CHIM 10127/28 7**	pET28a_ *Bsa*I_ CHIM 10127/28 6	Fo ⓅGAAGATTATCATGATTATATTTGACTCGAGCACCACCACCACC<br>Re ⓅTTTTTCTGCAACTGGAAAAGCTGGCTCGTCTC
**CHIM 10127/28 8**	pET28a_ *Bsa*I_NV10127	Fo ⓅAGTTTGTGGTGACTCGAGCACCACCACCACC<br>Re ⓅATGATAATCTTCTTTTTCTGCAAATGGAAAAGC
**CHIM 10127/28 9**	pET28a_ *Bsa*I_NV10127	Fo ⓅGATTATATTTGACTCGAGCACCACCACCACC<br>Re ⓅTTTTAAATCTACTTTTTCTGCAAATGG
**CHIM 10129/28 1**	pET28a_ *Bsa*I_NV10129	Fo CGTAAATATAGCATCAACTGCGGGCATAGTCAGCGG<br>Re CCGCTGACTATGCCCGCAGTTGATGCTATATTTACG
**CHIM 10129/28 2**	pET28a_ *Bsa*I_NV10129	Fo GCGGGCATAGTCAGCGGAGTCTTGCCCGTTTATTGCGC<br>Re GCGCAATAAACGGGCAAGACTCCGCTGACTATGCCCGC
**CHIM 10129/28 3**	pET28a_ *Bsa*I_CHIM 10128/29 2	Fo CGTAAATATAGCATCAACTGCGGGCATAGTCAGCGG<br>Re CCGCTGACTATGCCCGCAGTTGATGCTATATTTACG

SI Table 4. Molar extinction coefficients at 280 nm of purified proteins.

Protein	Molar extinction coefficient [M^{-1} cm^{-1}]
**NV10127**	23045
**NV10128**	20525
**NV10129**	20525
**CHIM 10127/28 1**	23045
**CHIM 10127/28 2**	21555
**CHIM 10127/28 3**	20525
**CHIM 10127/28 4**	21555
**CHIM 10127/28 5**	20525
**CHIM 10127/28 6**	21555
**CHIM 10127/28 7**	21555
**CHIM 10127/28 8**	24535
**CHIM 10127/28 9**	19035
**CHIM 10129/28 1**	20525
**CHIM 10129/28 2**	20525
**CHIM 10129/28 3**	20525
